# *Loa loa* Ecology in Central Africa: Role of the Congo River System

**DOI:** 10.1371/journal.pntd.0001605

**Published:** 2012-06-26

**Authors:** Louise A. Kelly-Hope, Moses J. Bockarie, David H. Molyneux

**Affiliations:** Centre for Neglected Tropical Diseases, Liverpool School of Tropical Medicine, Liverpool, United Kingdom; London School of Hygiene & Tropical Medicine, United Kingdom

## Background

*Loa loa* is a parasite that causes tropical eye worm, or Calabar swelling, a disease confined to tropical forests of Africa where it is transmitted by Tabanid flies of the genus *Chrysops* [1], [2]. Recent disease prevalence maps published by Zouré et al. [3] in *PLoS Neglected Tropical Diseases* highlight an unusual geographical distribution in the central African region, which may be related to distinct environmental or topographical features of the region. Using geographical information systems (GIS) and remotely sensed satellite data, we examined the broad geographical and ecological parameters and specific climate variables of *L. loa* to highlight factors that could affect or influence the distribution of *Chrysops* vectors, and the potential for transmission, and to explain this unique epidemiological pattern.

The filaria parasite *L. loa* has assumed increasing importance in recent years, as it is associated with severe adverse events (SAEs) when some individuals receive ivermectin during mass drug distribution programmes for the control of onchocerciasis [4]. Individuals with high *L. loa* microfilaremia in excess of 30,000 microfilaria/ml of blood have a higher risk of these adverse events, which are life-threatening unless treatment and care is available [5]. Patients display symptoms of a dysfunctional central nervous system manifested as coma as a result of encephalopathy, presumptively as a result of the rapid death of *L. loa* microfilariae [4]–[6], although the precise pathology remains unclear. Proper care usually results in full recovery, but in more remote areas access to effective care by formal health workers is often difficult with serious consequences [6].

*L. loa* was a neglected parasitic infection until the observations of its association with adverse events came to the attention of the African Programme for Onchocerciasis Control (APOC), the donor of ivermectin (Mectizan) (Merck & Co., Inc.), and the Mectizan Donation Programme. The recent paper by Zouré et al. [3] has defined the areas of high SAE risk following extensive surveys using the Rapid Assessment Procedure for Loiasis (RAPLOA) method, which is based on village surveys of a history of eye worm and assesses the potential risk of post-treatment *L. loa* encephalopathy [7]. High risk areas are those with a prevalence of over 40% of eye worm history. Earlier studies [8], [9] developed a spatial model to define high endemicity, which was based on ecological parameters, in particular the degree of forest cover given the association of the main vectors *Chrysops silacea* and *C. dimidiata* with moist broad leaf tropical forest habitat [1], [2], [10], as well as other potential environmental drivers of *Chrysops* ecology such as elevation and soil type.

The high prevalence of loiasis and the risks of SAEs [3] has been the major impediment to scaling up both the onchocerciasis [11] and lymphatic filariasis (LF) programmes [12] as both use ivermectin, which acts as a microfilaricide. The challenges of co-endemicity of the three filarial infections—*Onchocerca volvulus*, *Wuchereria bancrofti*, and *L. loa*—are especially important in countries such as the Democratic Republic of Congo (DRC) given its size and the number of people at risk. A recent paper [13] has reviewed the earlier literature on loiasis with particular reference to the DRC, and used a new micro-stratification overlap mapping (MOM) approach to demonstrate that there has been limited change in loiasis distribution over the last 50 years, that highly endemic areas coincided closely with vector distributions, and that *L. loa* was found in forested regions away from the main river system [13]–[15]. Given that the population in this region is sparse and has experienced limited change in human ecology, the current distribution of loiasis [3] is likely to be determined by well defined environmental, edaphic, or topographical features of the region.

## Mapping and Graphing Key Associations

To determine the broad geographical and ecological parameters of *L. loa*, we examined the distribution of the dense tropical forests, focussing on the extent of the Congo River system and the elevation and soil type in the region [16], [17], together with specific vegetation (Normalized Difference Vegetation Index, [NDVI]) [18], precipitation (mm), temperature (C°), and humidity (qa) variables [19], [20] in defined high and low loiasis areas.

**Figure 1:**
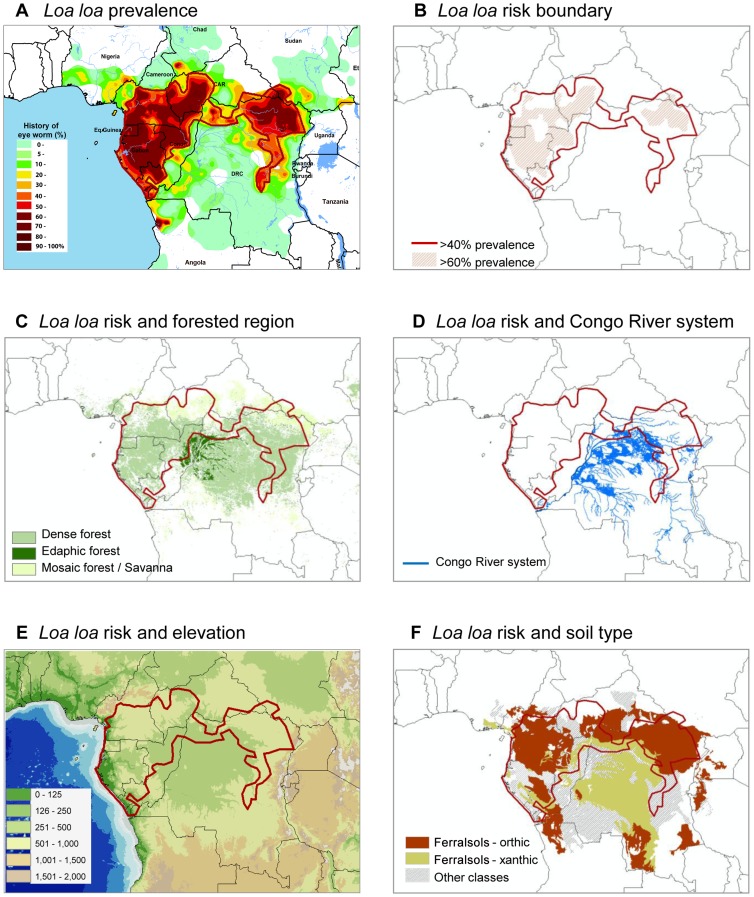
*L. loa* prevalence and risk in relation to topographical and ecological factors. (A, B) The loiasis map published by Zouré et al. [3] was imported into ArcGIS 9.3 (ESRI, Redlands, CA), and the high (>40%) and very high (>60%) prevalence areas digitised based on interpolated boundaries. (C, D, E, F) The relationship between disease risk and dense tropical forests, the Congo River and its tributaries, elevation, and soil type was examined using geo-referenced maps and data [16], [17].

The maps produced in Figure 1A and 1B show the unique shape of the high risk *L. loa* distribution across central Africa, with distinct east and west regions [3]. The distribution of tropical dense forests occurs in the same geographical region, and the high risk *L. loa* (>40%) boundaries (Figure 1B) were found to be within the limits of the tropical dense and mosaic savanna forests of Cameroon, Central Africa Republic, Congo, DRC, Equatorial Guinea, Gabon, and Sudan, but not the edaphic (flooded) forested areas of DRC (Figure 1C).

In contrast, the Congo River and its extensive tributaries were found predominately in the low risk *L. loa* region, where the river system extended >110,000 km, at elevations of <500 m, and is located in the centre of the tropical forested region and mainly in DRC (Figure 1D). The majority of the high risk *L. loa* regions were where fewer river systems (∼21,000 km in length) occurred at elevations between 500 and 1,000 m surrounding the Congo River system. Notably, the high risk western region showed more variability in elevation, and loiasis was lowest close to the Atlantic coast, whereas the high risk eastern region was bordered by very high elevations ranging from 1,000 to 2,000 m (Figure 1E), which geographically coincided with the outer limits of the tropical forests (Figure 1C) and different soil types (Figure 1F), a possible explanation of why loiasis was also not found in this region.

The main soil types found were classified as ferralsols, which are mineral soils that occur in tropical climates (Figure 1F). In the high risk *L. loa* regions, the dominant soil was orthic ferralsol, which is reddish brown in colour, fine-medium textured, usually occupying higher parts of undulating relief (slope 0%–30%) with a top soil composition of sand (29%), silt (18%), clay (53%), and nitrogen (0.22%). In contrast, in the low risk *L. loa* regions, the dominant soil was xanthic ferralsol, which is yellowish olive in colour, coarse textured, usually occupying lower parts of flat relief (slope 0%–8%) with a top soil composition of sand (52%), silt (8%), clay (40%), and nitrogen (0.09%). Other soil types in the study region included gleysols, nitosols, cambisols, and arenosols [17].

**Figure 2:**
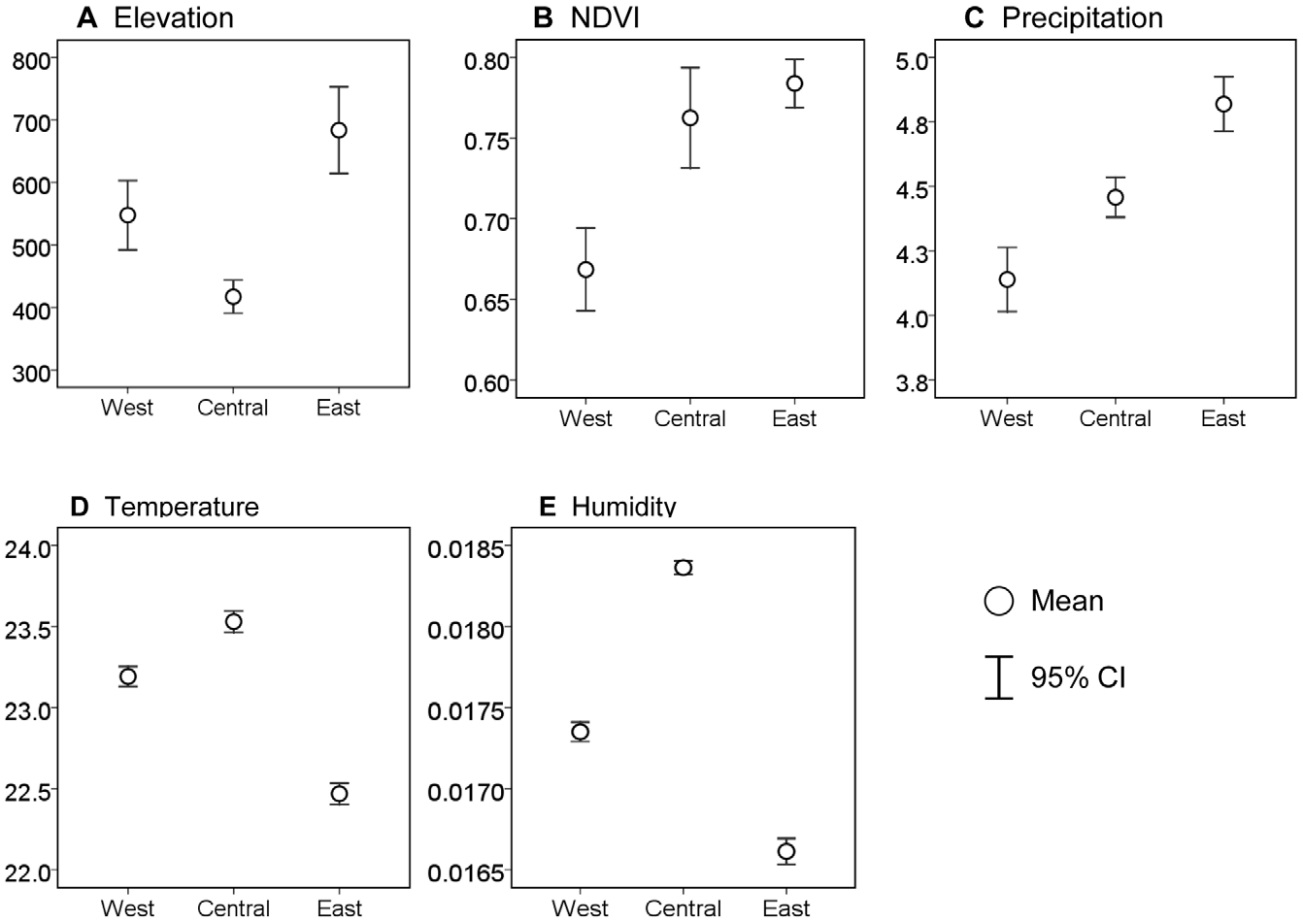
Comparison of environmental factors in high and low risk *L. loa* regions. (A–E) Environmental variables in very high (>60%) and low (<20%) prevalence areas were examined by randomly selecting 15 locations in the two high risk and one low risk areas (*n* = 45 in total). Underlying data on elevation (m), vegetation (NDVI), precipitation (mm), temperature (C°), and humidity (qa) [18]–[20] were extracted and analysed in PAWS Statistics 17.0 (SPSS, Inc., Chicago, IL).

Differences in elevation and climate variables between the east and west high risk *L. loa* regions (>60%) and central low risk *L. loa* region (<20%) are shown in Figure 2. Overall, comparisons indicate significant differences in elevation, temperature, and humidity (Figure 2A, 2D, 2E), with the two high risk regions recording significantly higher mean elevations of 548–684 m, and lower mean temperatures of 22.5°C–23.2°C, and lower mean humidity measures of 0166–0.174 qa, compared with the central low risk region with measures of 417 m, 23.5°C, and 0.0184 qa, respectively. Figure 2 also highlights NDVI to be significantly higher in the central region than the west region but not the east, and that all three regions had significantly different precipitation measures.

## The Congo River System and Control Priorities

This work shows clear geographical correlations between the recently published maps of high *L. loa* prevalence [3] and specific topographical and environmental parameters derived from various GIS tools and remotely sensed satellite data [16]–[20]. This broad perspective provides important insights into the general ecology of the disease, and factors potentially driving (or not) transmission.

The tropical dense and mosaic savanna forests are among the most important ecological determinants of *L. loa* as they are natural habitats of the main *Chrysops* spp. responsible for transmission [1], [2]. Several studies carried out in Cameroon, Congo, DRC, and Nigeria confirm that disease and vector infection rates are significantly higher in forest fringe or forested areas compared with other ecological settings such as grassland savanna, sunlit river banks, forest clearings, and villages [1], [14], [21]–[23]. Here we highlight that the extensive *L. loa* and SAE risk areas [3] occur within the limits of tropical forests of central Africa. However, we also highlight, perhaps more importantly, that there are vast forested regions with no or low *L. loa* prevalence where LF may be endemic [13], and hence the risks of SAEs during mass drug distribution when ivermectin is used are smaller than earlier assumed.

The presence of the extensive Congo River system in the middle of the tropical forest, overlapping in low *L. loa* prevalence areas that are predominately in the DRC [3], suggests that this distinctive topographical feature may inadvertently act as a natural barrier with definable environmental characteristics that are unfavourable to *Chrysops* spp. This is supported by Kelly-Hope et al. [13], who illustrated that historical *C. silacea* and *C. dimidiata* distributions [10] significantly overlapped with high *L. loa* prevalence with few *Chrysops* spp. recorded in the low-lying, low risk central area of the country. Thus, the Congo River system, which has one of the largest drainage basins in the world, the greater part covering more than 1 million km^2^ in the DRC, potentially provides protection against *L. loa* to millions of people living in this region.

The geography and ecology of the Congo River basin broadly differs from the surrounding areas that appear to be more suitable for transmission of *L. loa* (Figures 1 and 2). Notably, the east and west high risk loiasis regions had more mosaic savanna forest areas, fewer rivers, higher elevations, steeper slopes, medium silt/clay soils, and lower temperature and humidity levels than the central low risk region, where dense and edaphic (flooded) forests, flat relief, coarse soils, an extensive network of rivers, and hot humid conditions prevail. The extent to which these factors differentiate risk, and explain the lack of loiasis in the central region, is still unclear as no studies have specifically examined this. However, some insights may be gained from detailed studies on the bionomics of main *Chrysops* vectors [1], [23], [24].

Extensive field studies of the ubiquitous and efficient vector *C. silacea* indicate that specific environmental conditions are needed for optimal *L. loa* transmission [1], [23], [24]. This species lives in the forest canopy and has been shown to avoid deep shade and bright sunlight, tending to thrive in patchy light-shaded forest or fringe areas. It prefers to breed in slow running water, usually in mud covered in a few inches of water between nutrient rich decaying leaves in shaded undergrowth. Oviposition studies showed significant differences in the type of ground surface on which eggs were laid, with wet mud identified as the most important compared with hard, loose, or sandy soils where none were found [24]. Larvae appear to be attracted to the colour or brightness of leaves, and adult biting rates are highest in the cooler parts of the day when temperature and humidity levels are lowest [1], [23]. These biological and ecological factors, in part, may explain why loiasis prevalence is low in the Congo River basin region where vegetation is dense and shady, soils are predominately coarse, sandy and depleted in nutrients, the low-lying river system is subject to flooding [25], and hot humid conditions dominate year round [16]–[20].

Collectively, the maps and data presented in this paper provide an important large-scale perspective on the geographical and ecological drivers of *L. loa* transmission. This information may help planning appropriate and targeted control for loiasis and its *Chrysops* vectors. More research into alternative preventive chemotherapy and/or integrated vector management together with detailed epidemiological analysis of the original RAPLOA dataset will help the national onchocerciasis and LF programmes to move forward with their goals of disease control and elimination [11], [12].
